# First Case of Lung Abscess due to* Salmonella enterica* Serovar Abony in an Immunocompetent Adult Patient

**DOI:** 10.1155/2016/3159031

**Published:** 2016-06-26

**Authors:** Vassiliki Pitiriga, John Dendrinos, Emanuel Nikitiadis, Georgia Vrioni, Athanassios Tsakris

**Affiliations:** ^1^Department of Microbiology, Medical School, National and Kapodistrian University of Athens, M. Asias 75, Goudi, 11527 Athens, Greece; ^2^Metropolitan Hospital, Ethnarchou Makariou 9 & El. Venizelou 1, N. Faliro, 18547 Athens, Greece

## Abstract

In healthy individuals, nontyphoidal* Salmonella* species predominantly cause a self-limited form of gastroenteritis, while they infrequently invade or cause fatal disease. Extraintestinal manifestations of nontyphoidal* Salmonella* infections are not common and mainly occur among individuals with specific risk factors; among them, focal lung infection is a rare complication caused by nontyphoidal* Salmonella* strains typically occurring in immunocompromised patients with prior lung disease. We describe the first case of a localized lung abscess formation in an immunocompetent healthy female adult due to* Salmonella enterica* serovar Abony. The patient underwent lobectomy and was discharged after full clinical recovery. This case report highlights nontyphoidal* Salmonellae* infections as a potential causative agent of pleuropulmonary infections even in immunocompetent healthy adults.

## 1. Introduction

In developed countries, nontyphoidal* Salmonellae* (NTS) strains are a leading cause of self-limiting enterocolitis in healthy population; they are estimated to cause 94 million cases of gastroenteritis and 115,000 deaths globally each year [1]. Up to 5% of patients will develop secondary bacteremia [2], with low attributable mortality (1–5%). Localized extraintestinal infections develop as secondary complications in approximately 5–10% of cases with NTS bacteremia [3] and occur predominately in a wide variety of immunocompromised individuals [4], including patients with severe underlying diseases [5], immunocompromised elderly patients [6, 7], or children [8]. Mainly they involve the gastrointestinal tract, endothelial surfaces [9], pericardium [10], meninges [11], lungs, joints, and bones [12], or soft tissues. Among them, pleuropulmonary NTS infection is an infrequent manifestation [13, 14] mostly occurring in immunocompromised patients with prior lung or pleural pathology [15].

In healthy immunocompetent individuals, extraintestinal complications caused by NTS remain uncommon [16–19]. We present the first case of a lung abscess caused by* Salmonella* serovar Abony in an immunocompetent healthy young adult with no prior history of pulmonary disease or presence of any underlying disease.

## 2. Case Presentation

A 26-year-old female of Hellenic ethnicity was admitted to our outpatient clinic, reporting a 10-day history of low grade fever, chills, nausea, headache, urge to vomiting, and a dull pain over the right kidney area. Her past medical history was free of any chronic or acute infection or systemic disease, except for a prior admission to our hospital 1 year ago due to diarrhea not associated with any specific microorganism.

Her physical examination revealed temperature of 38°C, normal blood pressure (110/70 mmHg), and tachycardia (HR = 160 bpm). During palpation, a slight pain in right upper abdominal area was revealed with no other specific signs and symptoms. The respiratory examination was remarkable for dullness to percussion with decreased breath sounds over the lower right lung base. The remainder of the physical examination was unremarkable. Initial laboratory findings revealed a polymorphonuclear leucocytosis of 13.700/mm^3^ with 72% neutrophils and 18% lymphocytes, ESR of 29 mm/hr, and C-reactive protein of 5.31 mg/dL. No other pathological findings were indicated from the biochemical testing. Detailed investigations did not reveal any predisposing factors or evidence of an underlying immunodeficiency. More specifically, there was no evidence of malnutrition, no history of therapy with glucocorticoids or other immunosuppressive drugs, and no indication of immunoglobulin excess or deficiency through quantitative serum immunoglobulin tests, and blood tests were negative for chronic infections (HIV, viral hepatitis, etc.) or autoimmune disorders (antinuclear antibodies and other autoantibodies). Chest X-ray examination showed pneumonic infiltration in the lower one-third of the right hemithorax and laterally located dense appearance resembling left pleural effusion. Based on this evidence, the patient was diagnosed as having community-acquired pneumonia and after blood cultures were taken, antimicrobial treatment was initiated with intravenous ceftriaxone 1 gr/day + azithromycin 500 mg/day. Sputum, protected specimen brush (PSB) material of bronchial secretions, and three sets of blood specimens were also taken on admission for cultures which did not yield any pathogens.

Within the following two days, the patient's fever rose to 40°C despite the administration of antimicrobial therapy, and her condition deteriorated by developing dry cough, chest pain, total absence of breath sound during auscultation in right hemithorax, and dyspnea. Additionally, a strong right lumbar pain appeared. Three additional sets of blood cultures, taken while the patient was febrile, were negative.

**Figure 1:**
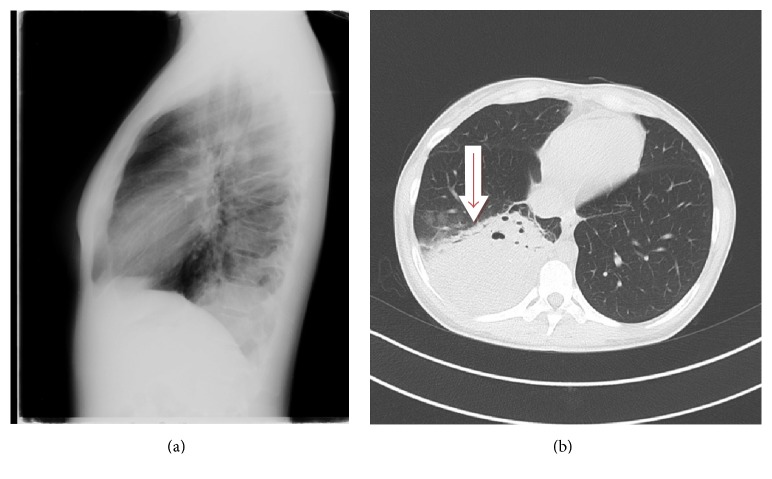
(a) Chest radiograph on presentation; (b) chest CT scanning on day 3 of admission, showing a large abscess in the right lower lobe.

On the third day, computed tomography (CT) scanning of the chest was performed and revealed a lung thick-walled abscess formation in the right lower lobe, with a surrounding inflammatory infiltrate, extended atelectasis, and pleural effusion to the right lower lobe. Figures 1(a) and 1(b) exhibit the size and morphology of the lung abscess. A subsequent ultrasound examination of the patient's liver, carried out in order to examine whether there was any subdiaphragmatic extension or origin of the infection, did not reveal any relevant evidence.

The combination of ceftriaxone + azithromycin was consequently discontinued and replaced by moxifloxacin 400 mg/day + tazobactam plus piperacillin (0.5 + 4.0) gr × 3/day + clindamycin 600 mg × 3/day. PCR for tuberculosis was performed and anti-*Echinococcus* IgG and IgM antibody titers were measured; however all the results were negative.

The next days, her fever did not subside, CRP levels rose gradually to 16.85 mg/dL, and the pleural effusion continued to rise. Blood cultures remained all negative for any bacterial growth.

On the sixth day of admission, the patient underwent lobectomy, owing to the lack of response to antibiotic therapy, the deterioration of symptoms, and the difficulty in approaching the specific lobe area by thoracentesis. Approximately 700 mL of pleural fluid was collected and sent to laboratory for biochemical analysis, Gram stain, cultures, and antimicrobial profile. Biochemical analysis of the pleural fluid showed the following: glucose of 74 mg/dL, lactate dehydrogenase of 730 U/L, total protein of 3.9 g/dL, and white blood cell count of 15.400/mm^3^ with 80% polymorphs. Gram staining and cultures of the pleural fluid were negative. In addition, the aspiration of the abscess revealed yellowish pus (about 45 mL) that was also sent for laboratory analysis the same day. Cultures of the pus sample collected from the abscess yielded a Gram-negative aerobic rod identified as* Salmonella enterica *subsp.* enterica* serovar Abony.* Salmonella* isolate was identified to the genus level by both the automated Vitek-2 System (bioMerieux, Inc., Hazelwood, MO) and the API 20E (bioMerieux, Inc., Hazelwood, MO). Serotyping of the isolate was performed using the somatic O and flagellar H antisera according to the Kauffman-White classification scheme (Difco Laboratories, Detroit, MI, USA). Molecular confirmation of* Salmonella* serotyping was carried out using the DNA microarray system Premi-Test® Salmonella (DSM Nutritional Products, Check-Points, Wageningen, Netherlands) [20]. Antimicrobial susceptibility testing was initially performed by the Vitek-2 System, according to the recommendations of the National Committee for Clinical Laboratory Standards [21] and confirmed by E-test (bioMerieux, Inc., Hazelwood, MO). The isolate was susceptible to commonly used antibiotics (ampicillin MIC of 0.75 *μ*g/mL, ceftriaxone MIC of 0.085 *μ*g/mL, cefotaxime MIC of 0.082 *μ*g/mL, ceftazidime MIC of 0.115 *μ*g/mL, ciprofloxacin MIC of 0.032 *μ*g/mL, moxifloxacin MIC of 0.016 *μ*g/mL, and trimethoprim-sulfamethoxazole MIC of 0.064 *μ*g/mL). Based on the laboratory report, the antimicrobial therapy was changed on the eighth day after admission, to sulfamethoxazole/trimethoprim (800 + 160) mg ×  2/day, moxifloxacin 400 mg/day, and clindamycin 600 mg × 3/day, along with supportive therapy.

The postoperative clinical condition of the patient improved noticeably. Six days after surgery, the patient's symptoms resolved and she was discharged on sulfamethoxazole/trimethoprim (800 + 160) mg × 2/day and ciprofloxacin 500 mg × 2/day, for 20 days.

## 3. Discussion

Even though the prevalence of invasive NTS in humans by means of bacteremia and extraintestinal infections is increasing worldwide among immunocompromised patients, particularly in developing countries, likely secondary to the high prevalence of coexisting malnutrition, malaria, and HIV infection, it remains uncommon in immunocompetent subjects [22]. Especially for NTS pleuropulmonary infection as a secondary manifestation in healthy individuals, only two reports exist in the literature documenting* Salmonella* Group B spp. as the primary cause of lung abscess in two immunocompetent female children [23, 24]. In another report of Genzen et al. [25], even though the 55-year-old man diagnosed with pulmonary* Salmonella* serovar Typhimurium infection was considered as immunocompetent, his medical history of chronic alcoholism and bronchitis should be taken into account as a significant predisposing factor for the invasive development of the infection. To our knowledge, this is the first report of lung abscess caused by NTS presenting in an immunocompetent healthy individual of the adult age group. Moreover, only one case report has been described in the literature documenting* Salmonella* serovar Abony to cause severe invasive disease, by means of disseminated intravascular coagulation, in an immunocompromised elderly patient [26]. We report for the first time an extraintestinal complication of lung abscess caused by* Salmonella* serovar Abony.

Lung infections by NTS may occur via several routes such as direct extension from a nearby infection, aspiration of gastric secretions, or hematogenous dissemination from the gastrointestinal tract. In the present case, our patient reported a history of diarrhea one year before the onset of illness. At that time, she was admitted to our clinic with a history of loose stools for a period of the prior ten days. She was given treatment with ciprofloxacin for three days before admission. On examination, she was not febrile and the stools were not accompanied by mucus or blood, while vomiting was absent. Stool and blood cultures were negative, possibly due to the antibiotic treatment and she was discharged 4 days after without identification of the infectious causative agent. This incident might be connected with the existing pulmonary complication, as it provides an indication of a potential gastrointestinal infection by* Salmonella *Abony with a subsequent seeding of the pathogen to the lungs through bacteremia.

## 4. Conclusion

This case report indicates that NTS strains should be considered as a potential etiological agent of infection in the differential diagnosis of pleuropulmonary infection causes, even among immunocompetent healthy adults.
